# Delivering a primary-level non-communicable disease programme for Syrian refugees and the host population in Jordan: a descriptive costing study

**DOI:** 10.1093/heapol/czaa050

**Published:** 2020-07-04

**Authors:** Éimhín Ansbro, Sylvia Garry, Veena Karir, Amulya Reddy, Kiran Jobanputra, Taissir Fardous, Zia Sadique

**Affiliations:** 1 Centre for Global Chronic Conditions, Department of Health Services Research and Policy, London School of Hygiene and Tropical Medicine, 15-17 Tavistock Place, London WC1H 9SH, UK; 2 Faculty of Epidemiology and Population Health, London School of Hygiene and Tropical Medicine, London WC1E 7HT, UK; 3 Médecins sans Frontières, Plantage Middenlaan 14 1018 DD Amsterdam, The Netherlands; 4 Médecins Sans Frontières, Lower Ground Floor, Chancery Exchange, 10 Furnival Street, London EC4A 1AB, UK; 5 Health Economy Directorate, Ministry of Health, Pr. Hamzah St., Amman, Jordan; 6 Department of Health Services Research and Policy, London School of Hygiene and Tropical Medicine, 15-17 Tavistock Place, London WC1H 9SH, UK

**Keywords:** Non-communicable disease, diabetes, hypertension, cardiovascular disease, humanitarian, conflict, cost, economic analysis, refugee, Syria, Jordan, programme

## Abstract

The Syrian conflict has caused enormous displacement of a population with a high non-communicable disease (NCD) burden into surrounding countries, overwhelming health systems’ NCD care capacity. Médecins sans Frontières (MSF) developed a primary-level NCD programme, serving Syrian refugees and the host population in Irbid, Jordan, to assist the response. Cost data, which are currently lacking, may support programme adaptation and system scale up of such NCD services. This descriptive costing study from the provider perspective explored financial costs of the MSF NCD programme. We estimated annual total, per patient and per consultation costs for 2015–17 using a combined ingredients-based and step-down allocation approach. Data were collected via programme budgets, facility records, direct observation and informal interviews. Scenario analyses explored the impact of varying procurement processes, consultation frequency and task sharing. Total annual programme cost ranged from 4 to 6 million International Dollars (INT$), increasing annually from INT$4 206 481 (2015) to INT$6 739 438 (2017), with costs driven mainly by human resources and drugs. Per patient per year cost increased 23% from INT$1424 (2015) to 1751 (2016), and by 9% to 1904 (2017), while cost per consultation increased from INT$209 to 253 (2015–17). Annual cost increases reflected growing patient load and increasing service complexity throughout 2015–17. A scenario importing all medications cut total costs by 31%, while negotiating importation of high-cost items offered 13% savings. Leveraging pooled procurement for local purchasing could save 20%. Staff costs were more sensitive to reducing clinical review frequency than to task sharing review to nurses. Over 1000 extra patients could be enrolled without additional staffing cost if care delivery was restructured. Total costs significantly exceeded costs reported for NCD care in low-income humanitarian contexts. Efficiencies gained by revising procurement and/or restructuring consultation models could confer cost savings or facilitate cohort expansion. Cost effectiveness studies of adapted models are recommended.

**Key Messages**

- Non-communicable disease (NCD) care is assumed to be expensive but studies of the costs of delivering primary-level NCD care are lacking in humanitarian settings and in low- and middle-income countries more broadly.
- This descriptive analysis of NCD care delivered in a humanitarian setting found that per patient per year cost ranged from INT$1424 to 1904, while cost per consultation ranged from INT$209 to 253.
- Costs were primarily driven by recurrent costs, especially drug and human resource costs, which increased in line with increasing programme complexity.
- Efficiency may be gained through adopting context-adapted drug procurement practices and via human resource redistribution.

## Background

Non-communicable diseases (NCDs) have been responsible for the majority of deaths worldwide for more than three decades, causing 71% (or 40.5 million) of the 56.9 million global deaths in 2016 (World Health Organization, 2018). NCDs accounted for 77% of mortality in pre-conflict Syria, led by cardiovascular disease (CVD; WHO, 2011). Following the prolonged conflict in Syria, now in its ninth year, almost 6.6 million refugees have fled, mainly into neighbouring countries; 670 000 refugees registered with the United Nations High Commissioner for Refugees (UNHCR) fled to Jordan. Irbid, Jordan’s second largest city, hosts over 165 000 refugees, the largest concentration after Amman (UNHCR, 2018a). Most live in urban settings, amongst the host community (UNHCR, 2018a). Previous studies confirmed the high burden of NCDs amongst Syrian refugees in Jordan (Doocy *et al.*, 2015, 2016) and Jordan’s public health system has been challenged to respond to this additional burden. Chronic diseases have traditionally been the remit of secondary and tertiary care in Jordan but national policy has more recently sought to increase primary care NCD capacity. Meanwhile, the humanitarian health system has supported the public health system response, adapting traditional camp-based care provision to serve urban-dwelling refugees (UNHCR *et al.*, 2014; UNHCR, 2018b; Akik *et al.*, 2019).

Médecins sans Frontières (MSF), a humanitarian medical organisation, supported the Jordanian health system in providing primary-level NCD care to Syrian refugees and the vulnerable host population in Irbid since 2014. Their programme involved a multi-disciplinary primary care model, which used context-adapted clinical guidelines; medications from the World Health Organization (WHO) Essential Medicines list; and task sharing, whereby tasks are redistributed to optimise staff and skill allocation. The service evolved to include specific mental health and psychosocial support (MHPSS) and a humanitarian support worker, who linked refugees to available social and protection services.

While there is a wealth of evidence on cost-effective, primary care-based clinical management of NCDs in stable high-income countries, there is limited evidence to guide the delivery of such interventions in low- and middle-income countries (LMICs), particularly for conflict-affected and forcibly displaced populations. The MSF institutional experience regarding NCD programming in humanitarian settings is equally limited (Miranda *et al.*, 2008; Ebrahim *et al.*, 2013).

Moreover, there has been limited focus on economic evaluations of health intervention in humanitarian crises (Makhani *et al.*, 2020). The sparse evidence on costs of NCD care from a patient perspective in humanitarian settings has largely been derived from self-reported household surveys rather than formal costing analyses. In Jordan, household surveys of urban-based Syrian refugees reported cost as the main barrier to accessing care for their NCDs (Doocy *et al.*, 2015; Rehr *et al.*, 2018). MSF provided free NCD consultations, medications and investigations; but patient accounts recorded as part of a programme evaluation corroborated the cost barriers faced when seeking NCD care for NCD conditions not covered by MSF or for specialist referral. Transport was reported as a barrier to accessing NCD care in several surveys, but MSF patients were reportedly willing to pay transport costs in order to access free care (Doocy *et al.*, 2015).

In addition, little is known about the costs from the *provider perspective* of delivering NCD care in humanitarian settings. Broad commentary on the expensive nature of NCD care has highlighted the perceived high cost of life-long and potentially complex management, and the immense strain placed on national healthcare systems by the influx of refugee populations with a high NCD burden (Spiegel *et al.*, 2010; UNHCR, 2014, 2015; Slama *et al.*, 2017; Boulle *et al.*, 2019). UNHCR has sought to address this by supporting NCD care at primary level and by exploring health insurance schemes for refugees (Guterres and Spiegel, 2012; UNHCR, 2014). To our knowledge, no costing studies describing provider or patient costs of NCD care in humanitarian settings have been published to date (Bischoff *et al.*, 2009; Spiegel, 2010; Spiegel *et al.*, 2010, 2014; Guterres and Spiegel, 2012; Demaio *et al.*, 2013; Jobanputra *et al.*, 2016; Slama *et al.*, 2017).

Limited available studies have focused on the high cost of statins to patients in the Eastern Mediterranean and its likely negative impact on adherence (Isma’eel *et al.*, 2012; UNRWA, 2018). Costing studies of NCD care in both LMICs and high-income countries point to drugs as high drivers of costs at community level (American Diabetes Association, 2013; Subramanian *et al.*, 2018), while the MSF experience across various settings confirms that human resources (HR) and medications tend to be the most expensive components of any programme. While there is a growing body of literature on market shaping strategies to contain rising healthcare costs, such as regional- or disease-specific pooled procurement mechanisms, there is little available evidence on the procurement practices of international non-governmental organisations (NGOs) (Huff-Rousselle and Burnett, 1996; WHO, 2007; Ewen *et al.*, 2014; USAID, 2014; Seidman and Atun, 2017; The Global Fund, 2017). This area may warrant exploration as these organisations engage further in the provision of chronic NCD care.

To contribute to evidence guiding humanitarian actors in tackling NCDs in complex settings, MSF undertook a mixed methods evaluation of the NCD programme in Irbid, north Jordan. Using the *RE-AIM* framework, we examined the programme's Reach, Effectiveness, Adoption (and acceptance) by patients and staff, and its Implementation and Maintenance over time, including the costs and fidelity of implementation. (Glasgow *et al.*, 2019). This article presents the costing component, describing the annual financial costs and major drivers of cost from the provider perspective. We also present sensitivity and scenario analyses performed around the major cost drivers (drug procurement and staffing) to explore optimisation of financial resources. Such data may help humanitarian organisations and other healthcare providers to design or adapt cost-effective interventions, and may have implications for the broader Jordanian health system response and scale up of primary-level NCD care.

## Methods

### Study context and intervention

MSF developed an NCD service for Syrian refugees and vulnerable members of the Jordanian host population at a Ministry of Health primary care clinic in Irbid in December 2014. Due to space limitations, a second city-centre site was opened within a local NGO clinic in April 2015. Both sites provided the same vertical services, i.e. they were not integrated into pre-existing activities at either site. They had the same staffing makeup, covered the same catchment area and shared the same management, training and supervision teams. In fact, both sites were amalgamated in 2019. By the end of the study period (the end of December 2017), 5045 patients had been enrolled; 30% were Jordanian, in keeping with government requirements.

The programme focused on NCDs and NCD risk factors responsible for the greatest mortality in pre-war Syria: hypertension, established CVD (angina, myocardial infarction, ischaemic stroke, transient ischaemic attack, peripheral vascular disease, congestive heart failure), diabetes types I and II, asthma and chronic obstructive pulmonary disease (COPD). It targeted those with pre-established relevant diagnoses or with new diagnoses made by MSF or referring services. Cancer care was excluded. MSF screened patients for other target NCDs and engaged in primary/secondary prevention via cardiovascular risk management, offering healthy living advice and drug therapy as appropriate. Among patients active by the end of 2017, ∼67% had hypertension, 60% had diabetes type II, 24% had CVD, 6% had asthma, 4% had diabetes type I and 2% had COPD, while over 70% had two or more target NCDs (internal MSF data).

Clinic-based care was initially provided by generalist doctors with the support of nurses, a health educator, a pharmacist and reception staff. In 2015, the service evolved with the addition of a family medicine specialist at each site and a home visit service with a dedicated doctor, nurse and driver. The home visit service was expanded and MHPSS counsellors and a humanitarian liaison officer were added in 2016, followed by a physiotherapist in 2017. Clinical staff were supported by an MSF project team in Irbid and a coordination team, including an epidemiologist, in Amman. Both included national and international administrative, logistical, management and clinical supervisory staff. The programme guidance stated that patients with uncontrolled disease should attend consultations monthly until stabilised and 3-monthly thereafter. Doctors performed most consultations. Task sharing to nurses of review appointments for stable patients was introduced in 2016, but nurses were performing only 6% of follow-up consultations by the end of 2017. Doctors continued to manage prescribing since nurses were not permitted to initiate or adjust medications by Jordanian law. Referrals were not funded by MSF and were excluded from cost calculations. Emergency cases were referred to the Jordanian public health service. Non-urgent referrals (most frequently ophthalmology, cardiology and nephrology) were made to public, private or other humanitarian providers. Referral patterns varied greatly over time as the availability of services, e.g. NGO-provided cardiac catheterisation, depended on short donor funding cycles. MSF capped the total cohort size at ∼4000 active patients to contain costs.

In many MSF settings, medications and supplies are imported via European-based procurement units e.g. Amsterdam Procurement Unit (APU). These command great purchasing power and can obtain NCD medications at competitive prices. Jordanian regulation, however, required international NGOs to purchase from the local market. MSF approved a number of Jordanian wholesale suppliers, which met MSF’s strict quality control criteria (MSF, 2016). Three MSF operational centres (Amsterdam, Paris and Barcelona) active in Jordan at the time of the study each procured medications separately, typically in 3–6 monthly order cycles. For drugs unavailable locally or with an excessive lead time, importation exceptions could potentially be granted by the Jordan Food and Drug Administration (Karir *et al.*, 2018).

### Cost analysis

This retrospective costing study was undertaken from the provider perspective, considering MSF as the provider. We used a combination of standard step-down and ingredients-based costing approaches, previously used in economic evaluations of health interventions in LMIC settings (Creese and Parker, 1994; UNAIDS, 2000; Terris-Prestholt *et al.*, 2010; Sweeney *et al.*, 2014). Given the detailed expenditure data available from MSF, we principally used step-down costing. This allocates overhead costs or resources in a step-wise fashion to all overhead departments and then to final cost centres (a unit that produces output and has a record of resource consumption, in this case, a clinical consultation) (Pavignani and Colombo, 2009). Ingredients-based costing requires the identification and specification of each resource component or input, used for delivering an individual service and the unit cost of each in order to calculate a total endpoint cost. In this case, we estimated how many minutes staff spent with patients during consultations, the time taken for supervision and on-job training and we utilised drug consumption data and unit costs.

Annual financial costs, i.e. those costs resulting from actual expenditure on goods and services, were calculated for the study period 2015–17. Economic costs (costs used by a programme that could have been productively used elsewhere) were not calculated, as there was no volunteer time or donated items, and the analysis took into account all resources used in delivering the programme. Thus, economic and financial costs would have been very similar.

### Data collection and management

A project timeline was developed with input from management staff. Information relating to the nature, location and mode of delivery of the NCD services was collected during a field visit in August 2017 by the lead investigator and was supplemented by informal interviews with medical supervisory staff. A data analysis tool was designed to collate and calculate the relevant financial costs by cost centre. Cost data were collected for the study period from the management and drug supply chain, including itemised annual expenditure data (Supplementary File S1).

Costs were categorised by service level (coordination, project and clinic level) and by programme output (Table 1). Overheads incurred at coordination level were allocated using a factor of 30%, derived from the mean estimate of the time coordination staff devoted to the Irbid NCD programme. Overheads from project and clinic level were allocated at 100%. 

**Table 1:** Overview of clinic outputs (number of active patients and consultations)

Year	2015	2016	2017
Total number of active patients at end of year (% increase from previous year)	2954	3656 (+24%)	3540 (−3%)
Number of consultations per year (% increase from previous year)	20 130	25 912 (+29%)	26 592 (+2%)

*Note*: The number of active patients and consultations increased as the clinic expanded to a second site to increase the service capacity. There was little change from 2016 to 2017 as the number of active patients was capped for operational reasons.

Coordination-level costs, involving the management team in Amman, were categorised into (1) capital, (2) recurrent (other than HR) and (3) HR costs. Project-level costs, involving the management team with administration and supervision functions in Irbid, and clinic-level costs, involving the combined costs of delivering clinical care at both clinic sites, were also classified into capital and recurrent costs and coded into specific categories. Specific start-up costs were not included. We considered that there were no administrative start-up costs since the pre-existing coordination team in Amman already had structures and supply chains in place. At project level, there was a 4-month lead-in period, involving the international team setting up the service and starting to enroll patients while gradually recruiting national staff. Costs incurred during this period were included as capital and recurrent costs, as appropriate. 

Capital costs included building works and purchase of biomedical equipment, office equipment, furnishings and vehicles whose nominal cost was >100 Euro (Creese and Parker, 1994). Capital costs were annualised using straight-line depreciation and given a lifespan of 20 years for building, 5 years for vehicles and 3 years for equipment (Creese and Parker, 1994).

Recurrent costs included HR (contracted staff salaries and insurance; temporary workers’ fees; experts’ visits); logistics (building rent, maintenance and operation; office supplies and furnishings); vehicle maintenance and operation; biomedical equipment and consumables; external laboratory costs; and drugs. *Ad hoc* training of clinical and administrative staff was included as a HR cost and was generally delivered by MSF supervisory staff and/or visiting experts from headquarters (Supplementary File S1). There was no formal start-up or refresher training. International staff salary, per diem and travel costs were attributed to the project personnel budget; international staff accommodation costs were attributed to project-level logistics costs. The MSF salary scales, activity data (e.g. operational reports) and discussion with management and clinical staff were used to understand costs regarding HR and activities.

Drug costs were analysed as a separate input, as they were anticipated to be a major driver of cost and thus a focus of sensitivity and scenario analyses. We used drug purchase inventories, clinic-level consumption data, average unit purchase prices provided by the MSF logistic team (available for 2016 and 2017 only) and the MSF standard procurement list of drug prices, the ‘Green List’. For 2016 and 2017, missing prices were substituted with the other year’s price, after appropriate inflation or deflation; deflated 2016 prices were used to calculate 2015 drug costs. Items categorised as drugs included medications and drug delivery systems dispensed to patients (e.g. spacer devices, glucometers, lancets, glucometer strips, insulin needles).

### Descriptive cost analysis

Data were analysed in Microsoft Excel. Costs were incurred in both Jordanian Dinar (JOD) and Euro (for non-drug items imported via APU and international staff costs). They were inflated to the base year 2017 and then converted to International Dollars (INT$) by dividing JOD by the general purchasing power parity (PPP) rate of 0.32 and Euro by 0.747 (OECD/Eurostat, 2012; OECD, 2017; World Bank 2018). The PPP index is recommended for comparing costs across countries as it adjusts for differences in relative prices between economies (Kanavos and Mossialos, 1999). The total annual cost of NCD clinical care was calculated for each year (2015, 2016 and 2017) by adding the allocated capital and recurrent costs incurred at clinic, project and coordination level. Major cost drivers were identified. Annual total drug cost and cost per drug were calculated. Endpoint costs were expressed as cost per patient active at the end of each year, and cost per consultation per year (using ‘total annual new and follow up medical consultations per year’ as the denominator).

### Scenario analyses

Multifactorial scenario analyses were performed around drug and personnel costs, the key drivers of total cost, to explore areas where greater cost efficiency might be gained. All were performed around 2017 base case costs.

We explored three hypothetical drug cost scenarios. The first involved importing all medications and related equipment from Europe via the APU, since this reflects the practice of MSF programmes in most other settings. We acknowledge its limited feasibility given strict regulation and import restrictions in Jordan (Supplementary File S2). Using the MSF Green list, specific items on the Irbid project medication list were substituted with clinically equivalent alternatives, and, in cases where multiple formulations were used in Irbid but only a single formulation was available from APU, we proposed purchasing the equivalent number of milligrams consumed in 2017 from APU (Supplementary File S3). The second, more feasible scenario, involved MSF negotiating the right to import a limited number of high-cost items. Focusing on the programme’s 20 most costly drug items (Supplementary File S4), we considered importing only items whose exact formulations were available from APU (*n* = 10). In both importation scenarios, 16% was added to cover international and national transport, taxes, import fees and storage costs (including cold chain, cargo release fees and rent of port storage), based on MSF logistics data and expert opinion (Karir *et al.*, 2018). A sensitivity analysis was performed to examine the impact of applying a minimum of 5% and maximum of 40% to this handling charge, using figures based on MSF expert opinion. The third, and likely most feasible, scenario involved leveraging potential purchasing power to negotiate competitive pricing with local suppliers. We estimated that a 20% price reduction could be achieved by: (1) joining with other MSF operating sections active in Jordan; (2) reducing order cycles to 6-monthly; and (3) working with a reduced number of suppliers.

Additional scenario analyses determined the impact on clinical staff salary costs of redistributing consultation activity among medical and nursing staff. These involved varying: (1) the proportion of follow-up consultations for stable patients that were task-shared to nurses from 6% (the level in December 2017) to 100%; (2) the proportion of the cohort classified as 'stable' from 60% (based on 2017 cohort data analysis) to 70% or 80%; (3) the size of the active cohort from 3540 (total active patients at the close of 2017) to a maximum of 5000. We did not assess the impact on *total* cost of increasing cohort size (i.e. the cost implications of purchasing and dispensing more medications). Each of the additional scenarios used the review frequency recommended in MSF guidelines: patients achieving clinical control were reviewed 3-monthly (4 times per year); new and uncontrolled patients were reviewed monthly (12 times per year). Based on data from other MSF NCD programmes, we assumed doctors reviewed all new and uncontrolled patients, while nurses performed consultations for controlled patients, referring an estimated 10% back for doctor review (Ansbro, 2018). Since nurses in Jordan are not permitted to initiate or adjust medications, we assumed 90% of patients reviewed by nurses remained stable and continued the same doctor-prescribed medication regime.

The Ethics Review Committee (Reference 12239) and the Ethics Review Board of the authors’ institutes granted ethical approval for the conduct of this study.

## Results

The total annual financial cost of the MSF Irbid NCD programme was 4–6 million INT$ with the absolute value increasing annually by 52% from INT$4 206 481 in 2015 to INT$6 400 611 in 2016 and by a further 5% to INT$6 739 438 in 2017 (Table 2). The large increase from 2015 to 2016 partly reflects the increasing number of patients enrolled during that period, facilitated by the addition of a second clinic site (Table 1). 

**Table 2:** Annual cost per cost category and endpoint costs for Irbid NCD Programme for 2015, 2016 and 2017

Year of programme		2015		2016		2017	
Type of cost		INT$^a^	Annual total (%)	INT$	Annual total (%)	INT$	Annual total (%)
Capital costs	Coordination-level capital investment^b^	2872	0.1	8029	0.1	10 160	0.2
	Clinical equipment and drug storage	22 883	0.5	29 105	0.5	33 447	0.5
	Building work and furnishings^c^	22 852	0.5	31 069	0.5	30 961	0.5
	Vehicle purchase^d^	0	0.0	32 166	0.5	32 166	0.5
	Total capital	48 606	1.2	100 369	1.6	106 733	1.6
Recurrent costs	Coordination costs (excl. HR^e^)	102 815	2.4	85 514	1.3	150 485	2.2
	Drugs	1 615 967	38.4	3 008 539	47.0	3 049 381	45.3
	Laboratory	360 054	8.6	478 186	7.5	445 169	6.6
	Biomedical equipment^f^	270 516	6.4	7272	0.1	6177	0.1
	Building rent, maintenance, utilities	260 254	6.2	313 152	4.9	370 681	5.5
	Recurrent transport costs^g^	65 379	1.6	129 515	2.0	40 076	0.6
	Staff costs including expert visit	1 477 885	35.1	2 269 379	35.5	2 553 894	37.9
	Human resources training	5006	0.1	8684	0.1	16 841	0.2
	Total recurrent	4 157 874	98.8	6 300 242	98.4	6 632 704	98.4
Total annual costs		4 206 481		6 400 611		6 739 438	
Endpoint costs							
Cost per patient per year^h^		1424		1751		1904	
Cost per consultation^i^		209		247		253	

a Costs are presented in 2017 International Dollars (using PPP to convert JOD and Euro nominal costs into INT$).

b Coordination capital investment includes purchase of office furnishings, IT equipment and vehicles; some remodelling work on the rented office in Amman.

c Building work and furnishings includes office furnishings, IT equipment and other large items, furniture, large building work costs for the project office and both clinic sites in Irbid.

d Vehicle purchase at project level.

e Includes all recurrent costs at coordination level (building rent, maintenance, transport, etc.) except for human resources (included in the human resources category).

f Recurrent biomedical equipment used in clinic, e.g. swabs, gloves, glucometer strips.

g Recurrent transport costs: vehicle operation and maintenance, fuel, taxi hire (other than to the international airport, which is included as an international staff cost).

h Cost per patient per year: based on total annual cost divided by total active number of patients at end of relevant year (see Table 1).

i Cost per consultation: based on total annual cost divided by total new plus follow-up medical consultations per year. It excludes individual health education or mental health sessions and group sessions.

The main cost drivers each year were drugs (38.4–47.0%) and HR (35.1–37.9%). Together, these accounted for 73.6–83.4% of total expenditure (Table 2). Most costs were recurrent (98.4–98.8%). Most cost categories accounted for a similar proportion of annual expenditure across years, although drug costs increased by 9% from 2015 to 2016. As expected, the majority of biomedical equipment expenditure occurred in the first year of operation, accounting for 6.4% of total costs in 2015 but only 0.1% in 2016 and 2017. The top 20 most costly medication and related equipment items are presented in Supplementary File S2. The most expensive item was Mixtard insulin, accounting for 14% of the total drug budget. Underlying data (Supplementary File S2) show that insulin products and related equipment accounted for 34% of the total drug budget while statins contributed 15% and inhalers and spacers 8%.

The per patient per year (PPPY) cost increased by 23% from 2015 to 2016 (INT$1424 to $1751). PPPY increased by a further 9% to INT$1904 in 2017 (Table 2). Similarly, the cost per consultation increased by 18% from 2015 to 2016 (INT$209 to INT$247) and by a further 3% to INT$253 in 2017.

**Figure 1:**
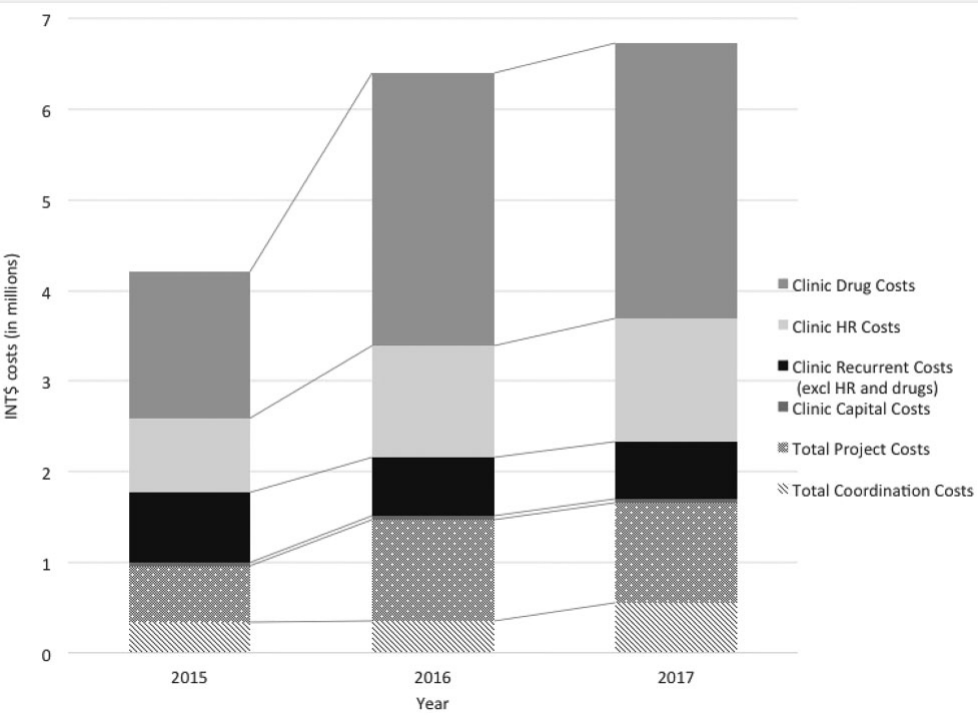
Annual cost per cost level for Irbid NCD Programme for 2015, 2016 and 2017, in International dollars.

The majority of costs were incurred at clinic level (75.2–77.2% of total costs each year), while field and coordination level costs accounted for a much lower proportion (14.8–17.4% and 5.6–8.1%, respectively) (Figure 1 and Supplementary File S5). Salaries, insurance and other costs required when employing Jordanian staff accounted for a fifth of the total budget. 

Table 3 presents several scenarios exploring alternative drug procurement arrangements. Scenario 1 outlines a hypothetical situation importing all medications and relevant equipment from the APU, which reflects the procurement model of many MSF programmes in other contexts. The total drug cost using this scenario was INT$962 076 (range: 870 845–1 161 127), representing a 68% saving on the base-case drug cost (62% at maximum import costs) or 31% of total costs. 

**Table 3:** Scenario analyses exploring options to reduce drug costs (INT$2017)^a^

	Base case (2017) (Table 2)	Scenario 1			Scenario 2			Scenario 3
		Import all drugs from Amsterdam Procurement Unit with various associated import costs (%)			Import 10 of most costly drugs items, available from MSF Essential Drugs List, with associated import costs (%)			Pooled procurement scenario^b^
		Min. (5%)	Expected (16%)	Max. (40%)	Min. (5%)	Expected (16%)	Max. (40%)	
Drug costs	3 049 381	870 845	962 076	1 161 127	2 116 757	2 155 316	2 239 444	2 439 505
Non-drug costs	3 688 844	3 688 844	3 688 844	3 688 844	3 688 844	3 688 844	3 688 844	3 688 844
Total annual cost	6 739 438	4 559 689	4 650 920	4 849 971	5 805 601	5 844 160	5 928 288	6 128 349
% Change vs base	0	−32%	−31%	−28%	−14%	−13%	−12%	−9%

a Costs are presented in 2017 International Dollars (using PPP to convert JOD and Euro nominal costs into INT$).

b The pooled procurement scenario involved pooling with other MSF sections active in Jordan, reducing the number of suppliers and reducing frequency of order cycles to 6-monthly.

Scenario 2 reflects a more realistic possibility for this specific context, whereby MSF would negotiate permission to import 10 of the top 20 most costly drug items. Significant savings of 29% of drug costs vs the base case (INT$894 065 or 13% of total costs) were still possible with this scenario, and were largely retained (27%; INT$809 937) at our estimated maximum import cost. For Scenario 3 we estimated, based on local expert opinion, that savings of 20% could be made compared with the local purchase prices obtained in 2017. This would result in potential savings of 9% of total programme costs.

Scenario analyses varying factors affecting work pattern are displayed in Table 4 (see also Supplementary File S6). The base case described patient load (3540 active patients) and staffing patterns as of the end of 2017, using salaries of currently employed doctors (two specialists and two non-specialists) and nursing time required for follow-up consultations of stable patients at 2017 rates (6%). 

**Table 4:** Scenario analysis varying work pattern and patient load

Variables	Base case	Scenarios			
	Current patient load and staffing	Scenario 1 Task sharing	Scenario 2 Task sharing with 70% controlled	Scenario 3 Task sharing with 70% controlled & cohort of 4500	Scenario 4 Task sharing 80% controlled and cohort of 5000
Cohort size	3540^a^	3540	3540	4500	5000
Proportion at clinical control	60%^b^	60%	70%	70%	80%
Specialist doctors^c^	2	1	1	1	1
Non-specialist doctors^c^^,^^d^	2	2.5	1.5	2	2.5
Nurses^c^	0.2	1.5	2	2.2^e^	2.8
Total annual salary cost^f^ (INT$ 2017)	307 528	288 208	246 376	311 387	302 457
% Change in cost vs base case	n/a	−6.3	−19.9	+1.3	−1.6

a Total number of active patients at end of 2017.

b Proportion of active cohort that is stable based on cohort analysis.

c Full-time equivalent.

d Figures rounded up to the nearest 0.5 of FTE.

e This scenario allowed for the dedication of an additional 0.2 FTE nurses to consultations vs Scenario B, who could be redeployed from other activities, such as triage and patient education.

f Annual total salary costs of doctors and nurses required to perform new and follow-up medical consultations.

Scenario 1 described the implications of adhering to guideline review intervals for the current cohort, categorising 60% as controlled. In this case, assuming only one specialist doctor was employed to manage the especially complex patients, 3.5 FTE (full-time equivalent) doctors and 1.5 FTE nurses were required, resulting in savings of 6.3% of clinical staff costs. Scenario 2 assumed all Scenario 1 parameters remained, but the proportion of controlled patients was increased to 70%, shifting more patients to 3-monthly nurse-led appointments. Thus, one FTE non-specialist doctor could be removed, while 0.5 FTE nursing time was added, resulting in clinical staff cost savings of 19.9% (INT$41 822). Scenario 3 proposed that the cohort could be increased by 1000 for almost the same cost as the base case (INT$311 387 vs 307 528) using the conditions of Scenario 2. Scenario 4 suggested that if the control rate could be increased to 80%, thereby shifting even more patients to 3-monthly nurse-led reviews, an almost 1500 extra patients could be added to the cohort for a slightly lower clinical salary cost than current base-case cost (−1.6%; INT$302 457 vs 307 528). Thus, clinical salary costs were most sensitive to the assumption that 70% of patients were achieving clinical control and were reviewed by a nurse on a 3-monthly basis. Clinical salary cost savings could be made with a similar sized cohort or, as in Scenarios 3 and 4, the cohort could be increased at a salary cost similar to the current 2017 base-case cost. Note, in these scenarios, we did not include the increased cost of drugs that would be incurred if the cohort size was increased.

## Discussion

To our knowledge, this is the first study to provide a detailed description of the costs of providing primary-level NCD care to Syrian refugees and the local population in the Middle East region, and one of the few to describe the costs of delivering NCD care in humanitarian settings globally. Our findings showed that total costs were primarily driven by drug and human resource costs and that most costs were incurred at the clinic level. Our scenario analyses indicated that the greatest cost efficiency could be gained by importing all medications from Europe, then by importing the top 10 most expensive items and, finally, by pooling procurement (in this case, between the various MSF operational centres). Less significant cost savings could be made through greater use of task shifting.

The total annual financial cost of delivering the MSF NCD programme in Irbid increased yearly from 2015 to 2017. This was due to increasing numbers of active patients over time but also to the delivery of a more complex programme requiring greater HR inputs. The year 2015 saw a gradual addition of staff and services, including the home visit service, the mental health service and additional counselling, pharmacy, medical and nursing staff (Ansbro, 2018). While a greater number of consultations was performed in 2017, they involved a smaller number of active patients, so fewer patients were seen more often, thereby reducing efficiency (Table 1).

From a cost structure perspective, costs other than drugs and HR contributed only one-fifth of the total. Of these, most were recurrent costs. Capital costs were minimal since MSF rented office and warehouse premises and space within pre-existing clinics.

Drugs were the major cost driver each year. As discussed, Jordan legislation requires NGOs to purchase drugs locally, unlike in many humanitarian contexts where NGOs can import drugs. The costs involved in insulin therapy (insulin, glucose reagent strips and lancets) featured prominently, despite insulin being prescribed at only 23% of visits in 2017. Atorvastatin accounted for 15% of the total drug budget in 2017, despite potential under-prescribing (only 25% of eligible patients were actually prescribed it) (Ansbro, 2018).

The majority of costs were incurred at clinic level, since the drug budget and clinical staff costs were allocated to this level. The costs associated with the highly qualified Jordanian medical, paramedical and support staff (salaries, insurance, medical costs) contributed approximately two-thirds of the HR budget. The total annual cost could be reduced by almost 25% (INT$1 657 960 in 2017) if the costs of MSF’s operational, logistical and medical supervisory support at central and local level were removed, reflecting potential savings if such a service were scaled up within a public healthcare system.

According to our scenario analyses, the total annual drug cost would be reduced by over two-thirds if MSF were to import all drugs from Europe at MSF warehouse prices (including import costs), potentially saving 31% (INT$2 087 305) of total programme costs. A more realistic scenario importing a limited number of costly items still resulted in drug cost savings of 12% of total costs. A 9% reduction in total costs (INT$609 876), obtained via the pooled procurement scenario, offered the least cost savings but may represent the most feasible option in the current regulatory environment.

Three pharmaceutical originator companies control 96% of the global insulin market. Significant work has been done to illuminate the global barriers and challenges in accessing affordable insulin (Beran *et al.*, 2016; Gotham *et al.*, 2018). Some humanitarian organisations have recently negotiated a reduced price per vial of human insulin from one originator company, which has introduced differential pricing for least developed countries, averaging 2.9 USD per vial in 2019 (Novo Nordisk, 2019). However, there is still significant advocacy and policy work to be done by WHO, humanitarian actors, governments, the research community and advocacy groups to address global disparities in insulin pricing and availability. In our analysis, underlying data show that MSF paid 9.81 JOD per vial in 2017 to local suppliers (30 INT$ using PPP or 13.83 USD using a direct currency conversion). Clearly, significant savings may be possible, either through negotiation with local insulin suppliers in Jordan or via importation. Echoing findings from other contexts, we also underline the significant additional costs associated with insulin therapy (glucometers, strips and lancets), which may also be amenable to negotiation with manufacturers or suppliers (Beran and Yudkin, 2010).

Our consultation delivery model scenario analyses demonstrated that these costs were more sensitive to frequency of patient review rather than to a change from doctor- to nurse-delivered consultations. As a greater proportion of patients were categorised as stable, incrementally greater cost efficiencies resulted, which could be translated into cost savings or to an expansion of the cohort within the same budget. Reducing review frequency of stable patients further still to 6-monthly would clearly result in further cost savings. These scenarios did not account for the time of other personnel directly involved in care delivery, such as pharmacists, health educators, triage nurses and reception staff, nor the increase in drug costs that would be incurred if the cohort size was increased (amounting to 861.41 INT$ annual per patient drug cost at 2017 base-case prices). Any reduction in HR costs, as demonstrated, would require significant restructuring of the programme, staff training and acceptance by patients, staff, within the local health system, legal and policy environment.

To our knowledge, there are no available published data to compare endpoint costs of primary-level NCD care delivery either in the Middle East region or in other humanitarian settings. Unpublished MSF data report *incremental* PPPY costs of INT$222 (2015) and INT$441 (2016), respectively, associated with adding diabetes care to pre-existing services in a chronic conflict setting in Mweso, Democratic Republic of Congo and with integrating NCD care with HIV and general outpatient services in Swaziland. However, comparisons must be made cautiously given different programme and procurement structures and local HR costs. A recent Kenyan study described patient-level direct annual costs of treatment for NCDs (hypertension, diabetes, asthma, COPD) at a quasi-public health facility (including data from MSF-Operational Centre Belgium Kibera Health Facility). Consultation fees, costs of medications and of admissions for acute exacerbations were included with total annual per patient costs ranging from $25.64 to $372.45 (USD 2015) (Subramanian *et al.*, 2018). The limited data on NCD care available from countries affected by the Syrian crisis focus on secondary- or tertiary-level care. A Turkish study showed that annual per patient cost for outpatient drugs and follow-up was 553.48 Lira (USD 121.38, 2015) for heart failure patients but the cost ingredients used were not reported (Aras *et al.*, 2016).

There are very limited available data to allow comparison of costs structures in the delivery of NCD care in LMIC or humanitarian settings. However, the unpublished MSF studies referred to above are consistent with this study in that HR and drugs accounted for the bulk of costs. The relatively high cost of insulin and related equipment has been found in previous studies. A review of medicine procurement processes and prices for drugs provided in UNRWA (United Nations Relief and Works Agency for Palestinian Refugees in the Near East) primary care clinics in 2010, prompted by budget constraints and the increasing demand for NCD drugs, underscored the high cost of anti-hypertensive and anti-diabetic medications, including insulin.

In the past, MSF and other humanitarian actors have tended to match their Essential Drug Lists to the WHO Essential Medications List and to set up parallel procurement systems, principally by importation from Europe and elsewhere. In addition, MSF has historically been less health system focused, and its exacting drug quality assurance (QA) standards can put it out of step with host country health systems. However, humanitarian NGOs, including MSF, increasingly provide services that are integrated within national health systems, especially in protracted crises. Thus, it may be more effective and ease procurement to match what is available in the local setting and to align with national health system procurement processes, especially when working in contexts with well-functioning health systems, such as the Middle East. Humanitarian NGOs may, therefore, need to modify their QA standards or to agree on a mutually acceptable QA approach with Ministries of Health. Furthermore, aligning with local prescribing practices, formulations and presentations (e.g. using individually boxed and branded medicines) may confer an added advantage in terms of acceptability to patients and local providers, as experience has shown that Syrian patients prefer to use drugs that are familiar to them (Ansbro, 2018; Garry *et al.*, 2018).

UNRWA procures most medications via central tender from pre-qualified suppliers (mostly located in Europe or the Middle East), while a minority of drugs are procured locally. In the review described earlier, UNRWA concluded that cost savings could be made through regular review of medication prices, competitive negotiation with a larger list of pre-qualified suppliers from a greater number of regions and via selective participation in Jordan’s Joint Procurement Department or the Gulf Cooperation Council effective pooled procurement tender processes (Ewen *et al.*, 2014; Seidman and Atun, 2017). MSF has also recently undertaken an in-depth assessment of access and affordability of NCD medications in Jordan and the region, which this article drew on, and concluded that savings could be made through pooled procurement by all MSF operational centres present in Jordan, by negotiation with local suppliers and by selective importation of expensive items . Perhaps the key lesson is that, given the high costs of providing chronic NCD drugs, humanitarian actors should undertake analyses of the pharmaceutical supply sector and should incorporate context-specific approaches to cost-effective procurement when designing or adapting NCD services.

### Limitations

This analysis did not examine direct costs from a patient perspective or indirect costs of NCDs in this population. Patient-level data were not examined in terms of service use. Each patient was treated the same regardless of diagnosis, date of entry to the cohort, duration of follow-up or whether an active or defaulting patient. Thus, costs could not be disaggregated by type of NCD or number/type of comorbidities, which may be an area for future research. Human resource costs for cadres other than doctors were based on staff estimates, rather than on formal staff time observation, which may have reduced the accuracy of these estimates. We did not include costs of external referral, which are not paid by MSF. In addition, given the specific Irbid programme model, separate start-up costs were not included but internal MSF training and epidemiologist support were. Wastage was not factored into drug costs. Other actors would need to take these elements into account if planning a similar programme.

Our scenario analyses around drugs are specific to the Jordan drug market and regulatory environment and may not be generalisable. However, we have illustrated that cost savings may be made by adapting procurement strategies to the local market. The HR-related scenario analyses include assumptions based on the local context or on other humanitarian contexts and may need to be adapted as appropriate. Finally, choosing to present costs in INT$ using PPP inflates the nominal JOD cost by a factor of three. Thus, costs may appear greater than if presented using the direct currency conversion of 1.41.

We suggest that future research should focus on (i) cost analyses from the patient perspective; (ii) prospective studies exploring provider costs on a per patient rather than aggregate basis, and (iii) on patient adherence and beliefs about medicines. We echo other authors’ suggestion that the WHO Regional Office for the Eastern Mediterranean would establish a regional procurement price database similar to that developed elsewhere (Ewen *et al.*, 2014).

## Conclusion

Cost estimates regarding the delivery of primary-level NCD care in humanitarian settings, and in LMICs more broadly, are lacking. Our study indicates that efficiency may be gained through adopting context-adapted procurement practices and via human resource redistribution. Our costing estimates will inform humanitarian actors in adapting this programme and in planning future NCD programmes in similar contexts. They may also have broader implications for the Jordanian health system response to the Syrian crisis and may inform policy makers scaling up primary-level NCD care in resource-constrained or crisis settings elsewhere.

## Supplementary Material

czaa050_Supplementary_Data

## References

[czaa050-B1] Akik C , Ghattas H, Mesmar S et al 2019. Host country responses to non-communicable diseases amongst Syrian refugees: a review. Conflict and Health 13: 8. 30949232 10.1186/s13031-019-0192-2 PMC6431037

[czaa050-B2] American Diabetes Association. 2013. Economic costs of diabetes in the U.S. in 2012. Diabetes Care 36: 1033–46. 23468086 10.2337/dc12-2625 PMC3609540

[czaa050-B3] Ansbro É. 2018. Mixed methods evaluation of MSF primary care based NCD service in Irbid, Jordan (2017-2018). London: MSF. Available at: http://fieldresearch.msf.org/msf/handle/10144/619309.

[czaa050-B4] Aras D , Aydoğdu S, Bozkurt E et al 2016. Cost of heart failure management in Turkey: results of a Delphi Panel. The Anatolian Journal of Cardiology 16: 554–62. 27515102 10.14744/AnatolJCardiol.2016.6999 PMC5368510

[czaa050-B5] Beran D , Ewen M, Laing R. 2016. Constraints and challenges in access to insulin: a global perspective. The Lancet Diabetes & Endocrinology 4: 275–85. 26857998 10.1016/S2213-8587(15)00521-5

[czaa050-B6] Beran D , Yudkin JS. 2010. Looking beyond the issue of access to insulin: what is needed for proper diabetes care in resource poor settings. Diabetes Research and Clinical Practice 88: 217–21. 20447710 10.1016/j.diabres.2010.03.029

[czaa050-B7] Bischoff A , Ekoe T, Perone N, Slama S, Loutan L. 2009. Chronic disease management in Sub-Saharan Africa: whose business is it? Int J Environ Res Public Health 6: 2258–2270 19742159 10.3390/ijerph6082258 PMC2738886

[czaa050-B8] Boulle P , Kehlenbrink S, Smith J, Beran D, Jobanputra K. 2019. Challenges associated with providing diabetes care in humanitarian settings. The Lancet Diabetes & Endocrinology 7: 648–56. 30878269 10.1016/S2213-8587(19)30083-X

[czaa050-B9] Creese A , Parker D. 1994. Cost Analysis in Primary Health Care: A Training Manual for Programme Managers. Geneva, Switzerland: World Health Organization.

[czaa050-B10] Demaio A , Jamieson J, Horn R, de Courten M, Tellier S. 2013. No n-communicable diseases in emergencies: a call to action. PLoS Currents. doi: 10.1371/currents.dis.53e08b951d59ff913ab8b9bb51c4d0de. 10.1371/currents.dis.53e08b951d59ff913ab8b9bb51c4d0de PMC3775888 24056956

[czaa050-B11] Doocy S , Lyles E, Akhu-Zaheya L et al 2016. Health service utilization among Syrian refugees with chronic health conditions in Jordan Wang Y (ed.). PLoS One 11: e0150088. 27073930 10.1371/journal.pone.0150088 PMC4830531

[czaa050-B12] Doocy S , Lyles E, Roberton T et al 2015. Prevalence and care-seeking for chronic diseases among Syrian refugees in Jordan. BMC Public Health 15: 1097. 26521231 10.1186/s12889-015-2429-3 PMC4628338

[czaa050-B13] Ebrahim S , Pearce N, Smeeth L et al 2013. Tackling non-communicable diseases in low- and middle-income countries: is the evidence from high-income countries all we need? PLoS Medicine 10: e1001377. 23382655 10.1371/journal.pmed.1001377 PMC3558465

[czaa050-B14] Ewen M , Al Sakit M, Saadeh R et al 2014. Comparative assessment of medicine procurement prices in the United Nations Relief and Works Agency for Palestine Refugees in the Near East (UNRWA). Journal of Pharmaceutical Policy and Practice 7: 13. 25379183 10.1186/2052-3211-7-13 PMC4210580

[czaa050-B15] Garry S , Checchi F, Cislaghi B. 2018. What influenced provision of non-communicable disease healthcare in the Syrian conflict, from policy to implementation? A qualitative study. Conflict and Health 12: 45. 30459826 10.1186/s13031-018-0178-5 PMC6233508

[czaa050-B16] Glasgow RE , Harden SM, Gaglio B et al 2019. RE-AIM planning and evaluation framework: adapting to new science and practice with a 20-year review. Frontiers in Public Health 7: 64. 30984733 10.3389/fpubh.2019.00064 PMC6450067

[czaa050-B17] Gotham D , Barber MJ, Hill A. 2018. Production costs and potential prices for biosimilars of human insulin and insulin analogues. BMJ Global Health 3: e000850. 10.1136/bmjgh-2018-000850 PMC6157569 30271626

[czaa050-B18] Guterres A , Spiegel P. 2012. The state of the world’s refugees: adapting health responses to urban environments. JAMA 308: 673–4. 22893161 10.1001/2012.jama.10161

[czaa050-B19] Huff-Rousselle M , Burnett F. 1996. Cost containment through pharmaceutical procurement: a Caribbean case study. The International Journal of Health Planning and Management 11: 135–57. 10172681 10.1002/(SICI)1099-1751(199604)11:2<135::AID-HPM422>3.0.CO;2-1

[czaa050-B20] Isma’eel H , Mohanna Z, Hamadeh G et al 2012. The public cost of 3 statins for primary prevention of cardiovascular events in 7 Middle East countries: not all of them can afford it. International Journal of Cardiology 155: 316–8. 22217486 10.1016/j.ijcard.2011.12.011

[czaa050-B21] Jobanputra K , Boulle P, Roberts B, Perel P. 2016. Three steps to improve management of noncommunicable diseases in humanitarian crises. PLoS Medicine 13: e1002180. 27824879 10.1371/journal.pmed.1002180 PMC5100924

[czaa050-B22] Kanavos P , Mossialos E. 1999. International comparisons of health care expenditures: what we know and what we do not know. Journal of Health Services Research & Policy 4: 122–6. 10387404 10.1177/135581969900400211

[czaa050-B24] Karir V, Bygrave H, Cepuch, C *et al* 2018. Accessibility to medicines for major non-communicable diseases in Jordan - 2018. (Internal briefing document). Geneva: Médecins sans Frontières (MSF) International. Available at: https://fieldresearch.msf.org/handle/10144/619475, accessed 2 October 2019.

[czaa050-B25] Makhani LA , Moran V, Sadique Z et al 2020. Examining the use of economic evaluations in health-related humanitarian programmes in low- and middle-income countries: a systematic review. Health Policy and Planning 35: 210–8. 31697373 10.1093/heapol/czz144

[czaa050-B26] Miranda JJ , Kinra S, Casas JP, Davey Smith G, Ebrahim S. 2008. Non-communicable diseases in low- and middle-income countries: context, determinants and health policy. Tropical Medicine & International Health: TM & IH 13: 1225–34. 18937743 10.1111/j.1365-3156.2008.02116.x PMC2687091

[czaa050-B27] MSF. 2016. *MSF Medical Product Qualification Scheme*. Geneva: Médecins Sans Frontières (MSF) International. Available at: https://www.msf.org/msf-medical-product-qualification-scheme, accessed 2 March 2019.

[czaa050-B28] Novo Nordisk. 2019. *Access to Insulin Commitment.* Copenhagen: Novo Nordisk. Available at: https://www.novonordisk.com/sustainable-business/commitment-to-access-and-affordability/our-access-to-insulin-commitment.html, accessed 31 October 2018.* *

[czaa050-B29] OECD. 2017. *Purchasing power parities (PPP) (indicator).* Paris: OECD.

[czaa050-B30] OECD/Eurostat. 2012. Eurostat-OECD Methodological Manual on Purchasing Power Parities (2012 Edition). Paris: OECD Publishing.

[czaa050-B31] Pavignani E , Colombo S. 2009. *Analysing Disrupted Helath Sectors: A Module Manual*. WHO: Module 6. Analysing Health Sector Financing and Ex. Geneva: WHO.

[czaa050-B32] Rehr M , Shoaib M, Ellithy S et al 2018. Prevalence of non-communicable diseases and access to care among non-camp Syrian refugees in northern Jordan. Conflict and Health 12: 33. 30008800 10.1186/s13031-018-0168-7 PMC6040066

[czaa050-B33] Seidman G , Atun R. 2017. Do changes to supply chains and procurement processes yield cost savings and improve availability of pharmaceuticals, vaccines or health products? A systematic review of evidence from low-income and middle-income countries. BMJ Global Health 2: e000243. 10.1136/bmjgh-2016-000243 PMC5435270 28589028

[czaa050-B34] Slama S , Kim H-J, Roglic G et al 2017. Care of non-communicable diseases in emergencies. The Lancet 389: 326–30. 10.1016/S0140-6736(16)31404-0 27637675

[czaa050-B35] Spiegel, P, Public Health and HIV Section at UNHCR. 2010. Urban refugee health: meeting the challenges. *Forced Migration Review: adapting to urban displacement. Issue 34.* University of Oxford Refugee Studies Centre, pp. 22–23. Available at: , accessed 18 June 2016.

[czaa050-B36] Spiegel P , Khalifa A, Mateen FJ. 2014. Cancer in refugees in Jordan and Syria between 2009 and 2012: challenges and the way forward in humanitarian emergencies. The Lancet Oncology 15: e290–7. 24872112 10.1016/S1470-2045(14)70067-1

[czaa050-B37] Spiegel PB , Checchi F, Colombo S, Paik E. 2010. Health-care needs of people affected by conflict: future trends and changing frameworks. The Lancet 375: 341–5. 10.1016/S0140-6736(09)61873-0 20109961

[czaa050-B38] Subramanian S , Gakunga R, Kibachio J et al; on behalf of the East African Economics and Implementation Group (EAEIG). 2018. Cost and affordability of non-communicable disease screening, diagnosis and treatment in Kenya: patient payments in the private and public sectors Larson BA (ed). PLoS One 13: e0190113. 29304049 10.1371/journal.pone.0190113 PMC5755777

[czaa050-B39] Sweeney S , Mosha JF, Terris-Prestholt F et al 2014. The costs of accessible quality assured syphilis diagnostics: informing quality systems for rapid syphilis tests in a Tanzanian setting. Health Policy and Planning 29: 633–41. 23894075 10.1093/heapol/czt049

[czaa050-B40] Terris-Prestholt F , Santos A, Sweeny S, Kumaranayake L. 2010. Guidelines for cost effectiveness analysis of syphilis screening strategies. In: **The Rapid Syphilis Test Toolkit: A Guide to Planning, Manag*e*ment, and Implementation.** London: London School of Hygiene and Tropical Medicine.

[czaa050-B41] The Global Fund. 2017. Global fund and pooled procurement mechanism (PPM). In: Jallow MT, Takayama M, Li (Roger) L (eds). *207 UN Business Seminar.* Geneva: The Global Fund. Available at: https://www.mofa.go.jp/mofaj/files/000268818.pdf, accessed 11 December 2018.

[czaa050-B42] UNAIDS. 2000. *Costing Guidelines for HIV Prevention Strategies*. Geneva: UNAIDS. Available at: http://data.unaids.org/publications/irc-pub05/jc412-costguidel_en.pdf, accessed 11 March 2020.

[czaa050-B43] UNHCR. 2014. UNHCR—*Update on Public Health and HIV/AIDS (EC/65/SC/CRP.16)*. Presented by The Deputy Director, Division of Programme Support and Management. Geneva: UNHCR.

[czaa050-B44] UNHCR. 2015. UNHCR—*Syria Regional Response Plan—Update*. Geneva: UNHCR.

[czaa050-B45] UNHCR. 2018a. *Situation Syria Regional Refugee Response*. Geneva: UNHCR.

[czaa050-B46] UNHCR. 2018b. *Health Access and Utilization Survey: Access to Healthcare Services among Syrian Refugees in Jordan. Geneva: UNHCR. Available at: https://data2.unhcr.org/en/documents/download/68539, accessed 6 September 2019.*

[czaa050-B47] UNHCR, UNFPA, IMC. 2014. *Population Based Health Access Assessment for Syrian Refugees in Non-Camp Settings Throughout Jordan: with Sub-Investigation on Non-Communicable Disease Management*. Geneva: Amman.

[czaa050-B48] UNRWA. 2018. *What We Do | UNRWA*. Amman: UNRWA. Available at: https://www.unrwa.org/what-we-do/services?program=39, accessed 23 April 2018.

[czaa050-B49] USAID. 2014. *Healthy Markets for Global Health: A Market Shaping Primer.* Washington: USAID. Available at: https://www.usaid.gov/cii/market-shaping-primer, accessed 11 December 2018.

[czaa050-B50] WHO. 2007. *Multi-country Regional Pooled Procurement of Medicines: Identifying Key Principles for Enabling Regional Pooled Procurement and a Framework for Inter-Regional Collaboration in the African, Caribbean and Pacific Island Countries*. Geneva, Department of Technical Cooperation for Essential Drugs and Traditional Medicine (TCM), Geneva: WHO.

[czaa050-B51] WHO. 2011. WHO | Noncommunicable Diseases Country Profiles 2011. Geneva: World Health Organization.

[czaa050-B52] World Bank. 2018. *Official Exchange Rate (LCU per US$, Period Average): Jordan, Euro Area | Data*. Washington: World Bank. Available at: https://data.worldbank.org/indicator/PA.NUS.FCRF?locations=JO-XC, accessed 10 April 2018.

[czaa050-B53] WHO. 2018. Global Health Estimates 2016: Disease Burden by Cause, Age, Sex, by Country and by Region, 2000-2016. Geneva: WHO.

