# Supplementary material for: Delivering a primary-level non-communicable disease programme for Syrian refugees and the host population in Jordan: a descriptive costing study
Source: Health Policy Plan. 2020 Jul 4;35(8):931–40. doi: 10.1093/heapol/czaa050 (PMC8312704; doi:10.1093/heapol/czaa050)
Supplement: czaa050_Supplementary_Data [file czaa050_supplementary_data.zip › czaa050-Suppl_Data/Supplementary File 1_final.docx]

Supplementary File 1: Summary cost data for the study period January 2015 to December 2017

| **Year of purchase** | **Type of cost** | **Level** | **Category** | **Description** | **Cost (JOD)** | **Cost (Euro)** | **Life expectancy / duration** |
| --- | --- | --- | --- | --- | --- | --- | --- |
| 2014 | Capital | Clinic Level | Drug storage | Fridges and temp control | 0.00 | 1552.39 | 3 |
|  |  | Project Level | Office equipment | Computers | 0.00 | 2642.00 | 3 |
| 2015 | Capital | Coordination Level | Office equipment | Furniture, office equipment, cold chain | 24668.17 | 0 | 3 |
|  |  |  | Vehicle purchase & maintenance | Vehicle purchase and parts | 233.56 | 0 | 5 |
|  |  | Project Level | Building and maintenance | Building construction and renovation | 0 | 0 | 20 |
|  |  |  | Vehicle purchase & maintenance | Vehicle purchase and parts | 0 | 0 | 5 |
|  |  |  | Furnishings | Chairs, desks, tables and filling cabinet, air con, fans, heating, curtains | 9632.04 | 0 | 3 |
|  |  |  | Office supplies | Computers, media, routers, screens and printers, long life batteries, curtains, cables | 364.00 | 5565 | 3 |
|  |  | Clinic Level | Building and maintenance | Construction and renovation | 5806.00 | 0 | 20 |
|  |  |  | Furnishings | Chairs, examination table, file cabinet, extension cables, air con, fans, heating | 6844.20 | 0 | 3 |
|  |  |  | Clinical equipment | BP cuff, stethoscope, otoscope, ECG machine, wheelchair, scale, spirometer, oxymeter, ophthalmoscope, defibrillator, cold chain | 19348.50 | 2802.12 | 3 |
| 2015 | Recurrent | Coordination Level | Coordination recurrent | All recurrent coordination activities (excluding HR) | 0 | 75911.09 |  |
|  |  |  | HR | HR costs | 0 | 169689.79 |  |
|  |  | Project Level | Training* | All costs associated with training, international and national staff | 1543.44 | 0 |  |
|  |  |  | Transport | All transport costs, except that directly associated with training | 17203.55 | 2794.58 |  |
|  |  |  | Office | All supplies for international staff house and office incl stationary, phones, clothes, utilities | 17394.05 | 1579.31 |  |
|  |  |  | Expert visit | Expert international visit | 391.30 | 0.00 |  |
|  |  |  | Staff costs | International staff costs - including relocation, per diem, medical costs, work permits | 15037.68 | 105469.63 |  |
|  |  |  |  | National staff salaries: domestic staff, coordination, admin, translator, logistics, driver, data operator | 68822.34 | 0 |  |
|  |  |  |  | Additional national staff costs: eg health insurance | 3126.25 | 0 |  |
|  |  |  | Building rent and maintenance | All building rental & maintenance costs – international staff house & office | 18725.30 | 0 |  |
|  |  | Clinic Level | Transport | Transporting patients only | 1788.50 | 0 |  |
|  |  |  | Drugs | All meds | 494348.78 | 0.00 |  |
|  |  |  | Biomedical equipment | Equipment used in clinic - e.g. swabs, gloves, glucometer strips | 82753.12 | 1577.87 |  |
|  |  |  | Lab | All lab tests (external) | 111020.50 | 0 |  |
|  |  |  | Office | Stationary and other supplies | 5745.60 | 0 |  |
|  |  |  | Building rent and maintenance | All building rental and maintenance costs - clinic | 37723.40 | 0 |  |
|  |  |  | Staff costs | National staff costs: eg health insurance | 12500.01 | 0 |  |
|  |  |  |  | Non-clinical salaries: registrar, health promotor, psychosocial | 50079.09 | 0 |  |
|  |  |  |  | Clinical salaries (pharmacists & assistants, nurses) | 110716.82 | 0 |  |
|  |  |  |  | Clinical salaries (doctors) | 80110.62 | 0 |  |
| 2016 | Capital | Coordination Level | Office equipment | Furniture, office equipment, cold chain | 55074.91 |  | 3 |
|  |  |  | Vehicle purchase & maintenance | Vehicle purchase and parts | 126.41 |  | 5 |
|  |  | Project Level | Building and maintenance | Building construction and renovation | 4118.00 | 0 | 20 |
|  |  |  | Vehicle purchase & maintenance | Vehicle purchase and parts | 49200.00 | 0 | 5 |
|  |  |  | Furnishings | Chairs, desks, tables and filling cabinet, air con, fans, heating, curtains | 2745.24 | 0 | 3 |
|  |  |  | Office supplies | Computers, media, routers, screens and printers, long life batteries, curtains, cables | 706.00 | 6078.13 | 3 |
|  |  | Clinic Level | Building and maintenance | Construction and renovation | 3300.00 | 0 | 20 |
|  |  |  | Furnishings | Chairs, examination table, file cabinet, extension cables, air con, fans, heating | 464.98 | 0 | 3 |
|  |  |  | Clinical equipment | BP cuff, stethoscope, otoscope, ECG machine, wheelchair, scale, spirometer, oxymeter, ophthalmoscope, defibrillator, cold chain | 3570.96 | 5176.14 | 3 |
| 2016 | Recurrent | Coordination Level | Coord recurrent | All recurrent coordination activities (minus HR) | 0 | 63290.16 |  |
|  |  |  | HR | HR costs | 0 | 196090.80 |  |
|  |  | Project Level | Training* | All costs associated with training, international and national staff | 2656.48 | 0 |  |
|  |  |  | Transport | All transport costs, except that directly associated with training | 39254.98 | 884.36 |  |
|  |  |  | Office | All supplies for international house and office incl stationary, phones, clothes, utilities | 26688.11 | 734.45 |  |
|  |  |  | Expert visit | Expert international visit | 0 | 0 |  |
|  |  |  | Staff costs | International staff costs - including relocation, per diem, medical costs, work permits | 27721.16 | 214039.69 |  |
|  |  |  |  | National staff salaries: domestic staff, coordination, admin, translator, logistics, driver, data operator | 114038.89 | 0 |  |
|  |  |  |  | Additional national staff costs: eg health insurance | 5298.36 | 0 |  |
|  |  |  | Building rent and maintenance | All building rental & maintenance costs – international staff house & office | 19196.48 | 0 |  |
|  |  | Clinic Level | Transport | Transporting patients only | 0 | 0 |  |
|  |  |  | Drugs | All meds | 920357.99 | 0.00 |  |
|  |  |  | Biomedical equipment | Equipment used in clinic - e.g. swabs, gloves, glucometer strips | 2067.75 | 379.79 |  |
|  |  |  | Lab | All lab tests (external) | 146284.50 | 0 |  |
|  |  |  | Office | Stationary and other supplies | 4229.55 | 0 |  |
|  |  |  | Building rent and maintenance | All building rental and maintenance costs - clinic | 45380.25 | 0 |  |
|  |  |  | Staff costs | National staff costs: eg health insurance | 20384.98 | 0 |  |
|  |  |  |  | Non-clinical salaries: registrar, health promotor, psychosocial | 61750.59 | 0 |  |
|  |  |  |  | Clinical salaries (pharmacists & assistants, nurses) | 174166.96 | 0 |  |
|  |  |  |  | Clinical salaries (doctors) | 121354.94 | 0 |  |
| 2017 | Capital | Coordination Level | Office equipment | Furniture, office equipment, cold chain | 15944.86 | 0 | 3 |
|  |  |  | Vehicle purchase & maintenance | Vehicle purchase and parts | 489.92 | 0 | 5 |
|  |  | Project Level | Building and maintenance | Building construction and renovation | 6154.90 | 0 | 20 |
|  |  |  | Vehicle purchase & maintenance | Vehicle purchase and parts | 0 | 0 | 5 |
|  |  |  | Furnishings | Chairs, desks, tables and filling cabinet, air con, fans, heating, curtains | 2096.85 | 120.02 | 3 |
|  |  |  | Office supplies | Computers, media, routers, screens and printers, long life batteries, curtains, cables | 3203.30 | 0 | 3 |
|  |  | Clinic Level | Building and maintenance | Construction and renovation | 2344.00 | 0 | 20 |
|  |  |  | Furnishings | Chairs, examination table, file cabinet, extension cables, air con, fans, heating | 0 | 0 | 3 |
|  |  |  | Clinical equipment | BP cuff, stethoscope, otoscope, ECG machine, wheelchair, scale, spirometer, oxymeter, ophthalmoscope, defibrillator, cold chain | 4727.25 | 266 | 3 |
| 2017 | Recurrent | Coordination Level | Coord recurrent | All recurrent coordination activities (minus HR) | 0 | 112412.64 |  |
|  |  |  | HR | HR costs | 0 | 287851.61 |  |
|  |  | Project Level | Training* | All costs associated with training, both international and national staff | 5389.20 | 0 |  |
|  |  |  | Transport | All transport costs, except that directly associated with training | 12295.32 | 67.50 |  |
|  |  |  | Office | All supplies for international staff house and office incl stationary, phones, clothes, utilities | 26365.78 | 1867.72 |  |
|  |  |  | Expert visit | Expert international visit | 388.00 | 0 |  |
|  |  |  | Staff costs | International staff costs - including relocation, per diem, medical costs, work permits | 27070.22 | 205634.74 |  |
|  |  |  |  | National staff salaries: domestic staff, coordination, admin, translator, logistics, driver, data operator | 138372.40 | 0 |  |
|  |  |  |  | Additional national staff costs: eg health insurance | 4124.27 | 0 |  |
|  |  |  | Building rent and maintenance | All building rental & maintenance costs – international staff house & office | 35827.00 | 0 |  |
|  |  | Clinic Level | Transport | Transporting patients only | 500.00 | 0 |  |
|  |  |  | Drugs | All meds | 975801.77 | 0 |  |
|  |  |  | Biomedical equipment | Equipment used in clinic - e.g. swabs, gloves, glucometer strips | 1976.75 | 0 |  |
|  |  |  | Lab | All lab tests (external) | 142454.05 | 0 |  |
|  |  |  | Office | Stationary and other supplies | 3341.25 | 0 |  |
|  |  |  | Building rent and maintenance | All building rental and maintenance costs - clinic | 52283.65 | 0 |  |
|  |  |  | Staff costs | National staff costs: eg health insurance | 14513.04 | 0 |  |
|  |  |  |  | Non-clinical salaries: registrar, health promotor, psychosocial | 44241.62 | 0 |  |
|  |  |  |  | Clinical salaries (pharmacists & assistants, nurses) | 229728.42 | 0 |  |
|  |  |  |  | Clinical salaries (doctors) | 147408.47 | 0 |  |

**Training: This category included transport, per diem and accommodation costs for Irbid project staff to attend internal MSF language, administrative and clinical training courses in Irbid or in Amman. In 2015, training costs included a 4-day health education course and a 3-day NCD clinical management course, each attended by 2 staff members. In 2016, UNHCR hosted a 6-day NCD management course attended by 3 MSF staff. In 2017, training costs included travel for 2 national staff to attend training in Amsterdam.*
