# Supplementary material for: Delivering a primary-level non-communicable disease programme for Syrian refugees and the host population in Jordan: a descriptive costing study
Source: Health Policy Plan. 2020 Jul 4;35(8):931–40. doi: 10.1093/heapol/czaa050 (PMC8312704; doi:10.1093/heapol/czaa050)
Supplement: czaa050_Supplementary_Data [file czaa050_supplementary_data.zip › czaa050-Suppl_Data/Supplementary File 2.docx]

Supplementary File 2: Rank of drug and related equipment items and proportion of total costs in 2017, in 2017 International Dollars.

| **Rank (2017)** | **Description** | **2017 Total Consumption** | **2017 INT$ cost** | **% total drug costs** |
| --- | --- | --- | --- | --- |
| 1 | INSULIN (MIXTARD) 100 IU, vial | 14399 | 441639.83 | 14.48 |
| 2 | STRIP, (glucometer accu check, blood glucose) | 361227 | 304164.42 | 9.97 |
| 3 | ATORVASTATIN, 20 mg, tab. | 453786 | 243895.79 | 8.00 |
| 4 | SYRINGE, s.u., Luer, insulin, 100 IU/1 ml + fixed needle | 461650 | 198495.07 | 6.51 |
| 5 | VALSARTAN, 80 mg, tab. | 501630 | 194115.13 | 6.37 |
| 6 | ENALAPRIL, 10 mg, tab. | 437117 | 164451.61 | 5.39 |
| 7 | SALMETEROL, 50mcg/FLUTICASONE 250mcg , 60 doses, diskus | 1496 | 148233.12 | 4.86 |
| 8 | AMLODIPINE, 5 mg, tab. | 304833 | 134345.62 | 4.41 |
| 9 | ATORVASTATIN, 80 mg, tab. | 47993 | 109484.03 | 3.59 |
| 10 | ATORVASTATIN, 10 mg, tab. | 315412 | 108422.88 | 3.56 |
| 11 | SALMETEROL, 50mcg/FLUTICASONE 500mcg , 60 doses, diskus | 695 | 93935.05 | 3.08 |
| 12 | OMEPRAZOLE, 20 mg, gastro-resistant caps. | 204397 | 83036.28 | 2.72 |
| 13 | GLIBENCLAMIDE, 5 mg, breakable tab. | 742992 | 78432.09 | 2.57 |
| 14 | ATENOLOL, 50 mg, tab. | 232239 | 57580.76 | 1.89 |
| 15 | METFORMIN hydrochloride, 850 mg, tab. | 639820 | 54564.65 | 1.79 |
| 16 | HYDROCHLOROTHIAZIDE, 25mg, tab. | 229850 | 47112.07 | 1.54 |
| 17 | ENALAPRIL maleate, 20 mg, tab. | 75596 | 45709.59 | 1.50 |
| 18 | VALSARTAN, 160 mg, tab. | 108345 | 45034.28 | 1.48 |
| 19 | ENALAPRIL maleate, 5 mg, tab. | 149569 | 44211.66 | 1.45 |
| 20 | RANITIDINE, 75 mg, tab. | 202588 | 35934.05 | 1.18 |
| 21 | BISOPROLOL fumarate, 5 mg, tab. | 164055 | 35271.83 | 1.16 |
| 22 | ACETYLSALICYLIC acid (aspirin), 100mg, tab | 578274 | 33160.40 | 1.09 |
| 23 | INSULINE Pen 30-70, flex pen , 300 IU | 1359 | 30959.72 | 1.02 |
| 24 | FUROSEMIDE, 40 mg, tab. | 180360 | 28000.89 | 0.92 |
| 25 | FERROUS SULFATE, 80 mg, tab. | 102110 | 25527.50 | 0.84 |
| 26 | INSULIN HUMAN, ISOPHANE (NPH) 100 UI/ml, 10 ml, vial N | 776 | 23789.25 | 0.78 |
| 27 | AMLODIPINE, 10 mg, tab. | 85912 | 21547.80 | 0.71 |
| 28 | METFORMIN hydrochloride, 500 mg, tab. | 297500 | 19188.75 | 0.63 |
| 29 | AMOXICILLIN 875mg / CLAVULANIC acid 125mg, tab. | 9976 | 16271.17 | 0.53 |
| 30 | SALBUTAMOL sulfate, eq.0.1mg base/puff, 200 puffs, aerosol | 3069 | 16121.07 | 0.53 |
| 31 | PARACETAMOL (acetaminophen), 500 mg, tab. | 252982 | 15811.38 | 0.52 |
| 32 | ISOSORBIDE DINITRATE, 20 mg, tab. | 78285 | 12895.01 | 0.42 |
| 33 | GLUCOMETER, Accu Check blood glucose monitor | 192 | 10546.24 | 0.35 |
| 34 | THYROXINE 50 mcg tab | 165647 | 10057.88 | 0.33 |
| 35 | ALLOPURINOL, 100 mg, tab. | 80562 | 9727.86 | 0.32 |
| 36 | SPIRONOLACTONE, 25 mg, tab. | 26755 | 9202.88 | 0.30 |
| 37 | METRONIDAZOLE, 500 mg, tab. | 1460 | 9033.75 | 0.30 |
| 38 | INSULIN HUMAN, RAPID 100 IU/ml, 10 ml, vial N | 257 | 7882.59 | 0.26 |
| 39 | AMITRIPTYLINE hydrochloride, 25 mg, tab. | 41840 | 7391.30 | 0.24 |
| 40 | CLOPIDOGREL 75 mg, tab. | 21970 | 7084.64 | 0.23 |
| 41 | AUTOINJECTOR NEEDLE, 31G, 0.25 x 6mm | 12940 | 6584.03 | 0.22 |
| 42 | RANITIDINE, 300 mg, tab. | 14350 | 6247.18 | 0.20 |
| 43 | FLUTICASONE, 50mcg, 120 doses, Evohaler | 263 | 6056.75 | 0.20 |
| 44 | SPACER, 155 ml with mask 6yrs- adult + mouthpiece | 42 | 5775.00 | 0.19 |
| 45 | BECLOMETASONE dipropionate, 0.25mg/puff, 200 puffs, aerosol | 206 | 5401.06 | 0.18 |
| 46 | AZITHROMYCIN, 250 mg, caps. | 1326 | 4696.64 | 0.15 |
| 47 | MICONAZOL nitrate, 2%, cream, 30 g, tube | 1048 | 3867.78 | 0.13 |
| 48 | IPRATROPIUM INHALER 20 MCG/PUFF (AS BROMIDE) 200 DOSE PER BOTTLE | 205 | 3680.05 | 0.12 |
| 49 | BLOOD LANCET for lancing device | 69160 | 3468.81 | 0.11 |
| 50 | WARFARIN 5 mg | 23855 | 2981.88 | 0.10 |
| 51 | DIGOXIN, 0.25 mg, tab. | 17348 | 1984.72 | 0.07 |
| 52 | FLUCONAZOLE, 50 mg, caps. | 266 | 1857.20 | 0.06 |
| 53 | CIPROFLOXACIN hydrochloride, eq. 500 mg base, tab. | 3380 | 1816.64 | 0.06 |
| 54 | CEFIXIME, 400 mg, tab. | 550 | 1770.31 | 0.06 |
| 55 | AMOXICILLIN, 500 mg, caps. | 6450 | 1420.21 | 0.05 |
| 56 | BISACODYL, 5 mg, tab. | 9880 | 1345.22 | 0.04 |
| 57 | ISOSORBIDE DINITRATE, 5 mg, sublingual tab. | 21330 | 1333.13 | 0.04 |
| 58 | NEOMYCIN and BACITRACIN, 5 MG + 25 IU cream | 309 | 1289.87 | 0.04 |
| 59 | PREDNISOLONE, 5 mg, tab. | 17375 | 1077.25 | 0.04 |
| 60 | HYDROCORTISONE acetate, 1%, ointment, 15 g, tube | 320 | 1020.00 | 0.03 |
| 61 | CHLORPHENAMINE maleate, 4 mg, tab. | 16308 | 1019.25 | 0.03 |
| 62 | HYOSCINE BUTYLBROMIDE (scopolamine butylbromide), 10 mg, tab | 2560 | 935.60 | 0.03 |
| 63 | IBUPROFEN, 400 mg, tab. | 9728 | 635.99 | 0.02 |
| 64 | SPACER, 155 ml with mask 18m-5yrs + mouthpiece | 3 | 412.50 | 0.01 |
| 65 | COTRIMOXAZOLE, 400 mg / 80 mg, tab. | 650 | 125.78 | 0.00 |
| 66 | NYSTATIN, 100,000 IU/ml, oral susp. | 12 | 79.24 | 0.00 |
| 67 | SODIUM chloride, 0.9%, 500 ml, flex. bag, PVC free | 12 | 78.93 | 0.00 |
| 68 | SALBUTAMOL, solution for nebulizer, 20ml | 5 | 51.32 | 0.00 |
| 69 | PARACETAMOL (acetaminophen), syrup, 125 mg/5ml, 100ml bot. | 6 | 48.00 | 0.00 |
| 70 | SODIUM chloride, 0.9%, 100 ml, flex. bag, PVC free | 9 | 29.60 | 0.00 |
| 71 | IPRATROPIUM bromide, 250mcg/ml, 5ml, vial | 10 | 8.28 | 0.00 |
| 72 | DEXTROSE (GLUCOSE), 5%, 500 ml, flex. bag, PVC free | 2 | 6.58 | 0.00 |
| 73 | HYDROCORTISONE sodium succinate, eq.100mg base, powder, vial | 7 | 5.84 | 0.00 |
| 74 | DICLOFENAC sodium, 25 mg/ml, 3 ml, amp. | 0 | 0.00 | 0.00 |
| 75 | METOCLOPRAMIDE hydrochloride, 5 mg/ml, 2 ml, amp. | 0 | 0.00 | 0.00 |
| 76 | AMOXICILLIN 500 mg / CLAVULANIC acid 125 mg, tablet | 0 | 0.00 | 0.00 |
| 77 | CARBAMAZEPINE, 200 mg, tab. | 0 | 0.00 | 0.00 |
| 78 | CEFIXIME, 200 mg, tab. | 0 | 0.00 | 0.00 |
| 79 | FLUOXETINE hydrochloride, eq. 20 mg base, caps. | 0 | 0.00 | 0.00 |
| 80 | HALOPERIDOL, 5 mg, tab. | 0 | 0.00 | 0.00 |
| 81 | LPV 200 mg / r 50 mg, tab. | 0 | 0.00 | 0.00 |
| 82 | METOCLOPRAMIDE HYDROCHLORIDE, 10 mg, tab. | 0 | 0.00 | 0.00 |
| 83 | METHYLDOPA, 250 mg, tab. | 0 | 0.00 | 0.00 |
| 84 | PAROXETINE, 20 mg, breakable tab. | 0 | 0.00 | 0.00 |
| 85 | PHENOXYMETHYLPENICILLIN, 1000 mg, tab. | 0 | 0.00 | 0.00 |
| 86 | RISPERIDONE, 1 mg, tab. | 0 | 0.00 | 0.00 |
| 87 | TDF 300 mg / 3TC 300 mg, tab. | 0 | 0.00 | 0.00 |
| 88 | VALPROATE SODIUM, 200 mg, gastro-resistant tab. | 0 | 0.00 | 0.00 |
| 89 | AZT 300 mg / 3TC 150 mg, tab. | 0 | 0.00 | 0.00 |
| 90 | GABAPENTIN, 400 mg, cap. | 0 | 0.00 | 0.00 |
| 91 | VACCINE HEPATITIS B, 1 adult dose, monodose vial | 0 | 0.00 | 0.00 |
| 92 | GABAPENTIN, 100 mg, cap. | 0 | 0.00 | 0.00 |
| 93 | CALCIUMcarbonate,eq.500mgCa,tab | 0 | 0.00 | 0.00 |
| 94 | Pantoprazole 20 mg tab. | 0 | 0.00 | 0.00 |
| 95 | CHLORAMPHENICOL, 0.5%, eye drops, sterile, 10 ml, bot. | 0 | 0.00 | 0.00 |
| 96 | TETRACYCLINE hydrochloride, 1%, eye ointment, ster, 5g, tube | 0 | 0.00 | 0.00 |
| 97 | CEFTRIAXONE sodium, eq. 1 g base, powder, vial | 0 | 0.00 | 0.00 |
| 98 | EPINEPHRINE (adrenaline) tartrate, eq.1mg/ml base,1ml amp IM | 0 | 0.00 | 0.00 |
| 99 | FUROSEMIDE, 10 mg/ml, 2 ml, amp. | 0 | 0.00 | 0.00 |
| 100 | CHLORPHENIRAMINE MALEATE, 10 mg/ml, 5 ml amp. | 0 | 0.00 | 0.00 |
| 101 | SALBUTAMOL, solution for nebulizer, 20ml | 0 | 0.00 | 0.00 |
| 102 | CLARITHROMYCIN 125mg/5ml, granules for susp, 60 ml bot | 0 | 0.00 | 0.00 |
|  | **Total Drug Cost** |  | **3049380.53** | **100** |
|  | Total insulin |  | 504271.39 | 16.54 |
|  | Total insulin AND related equipment |  | 1027529.97 | 33.70 |
|  | Total statin |  | 461802.70 | 15.14 |
|  | Total inhaler and spacer device |  | 258092.47 | 8.46 |
|  |  |  |  |  |
