# Supplementary material for: Delivering a primary-level non-communicable disease programme for Syrian refugees and the host population in Jordan: a descriptive costing study
Source: Health Policy Plan. 2020 Jul 4;35(8):931–40. doi: 10.1093/heapol/czaa050 (PMC8312704; doi:10.1093/heapol/czaa050)
Supplement: czaa050_Supplementary_Data [file czaa050_supplementary_data.zip › czaa050-Suppl_Data/Supplementary File 3.docx]

*Supplementary* *File 3: Decisions made for Scenario Analysis importing all drugs from Amsterdam Procurement Unit*

| **Item on Jordan OCA Mission drug list** | **Replaced with Item on MSF Green List 2017** | **Equivalent dosage** |
| --- | --- | --- |
| Bacitracin/ Neomycin 5g cream | Chloramphenicol eye drops | (1 x10 ml bottle) |
| Insulin mixtard 100 unit vial | Insulin (30/70) / ml 10 ml vial | 1 vial for 1 vial |
| Ferrous sulphate 80 mg | Ferrous salt 65 mg | 1 tablet for 1 tablet |
| Pen V 1000 mg | Pen V 250mg x 4 | 4 tablets for 1 tablet |
| Ranitidine 75 mg | Ranitidine 100 mg (but purchase equivalent total mgs) | Purchase equivalent number of mg from MSF |
| Clarithromycin suspension 1 bottle | Azithromycin suspension 1 bottle | 1 bottle |
| Valsartan 80 mg | Losartan 50 mg | 1 tablet for 1 tablet |
| Metformin 850mg | Metformin 500mg | Purchase equivalent number of mg from MSF |
| Atrovent nebuliser | Atrovent nebuliser | 5ml nebuliser = 2 x 2ml nebuliser |
| Chlorphenamine IV | Promethazine IV | 1 vial for vial |

If the same drug was not available, then a clinically equivalent drug and dose was selected (as listed in the table above).

If the same drug but different formulation/dose was available for a particular drug, the general rule was to purchase the equivalent number of mg from MSF stores. If the dose was lower on the Irbid drug list, then we purchased the same number of milligrams of the drug (i.e. higher MSF dose x lower consumption figure to equivalent number of milligrams consumed). If dose is higher on Irbid list, multiplied dose available on MSF list to give equivalent number of mg (and therefore multiply the price proportionally) e.g. Metformin 850 mg on Irbid list x consumption = number mg to purchase from MSF; divide by 500 = number of MSF tabs to purchase.

If the item on the Jordan Mission drug list was a suspension, we changed the antibiotic to a clinically appropriate replacement and purchased the equivalent of a bottle (i.e. one course).

The same was done for combination inhalers. We purchased the clinical equivalent of a monthly course of the separate inhaled ingredients. For a single ingredient inhaler, we purchased a monthly course at clinically equivalent steroid dose.

Allopurinol was not available nor any equivalent on the MSF Green List. This was replaced with aprice from WHO International Drug Price Indicator Guide online and was converted into EUR from US$ (2015) mshpriceguide.org for the original calculations.

Insulin pen. The closest available to Insulin 30/70 3ml autoinjector pen on the Jordan Mission Drug List was the Insulin Lispro 25/75 autoinjector 3 ml pen on the MSF Green List.
