# Supplementary material for: Delivering a primary-level non-communicable disease programme for Syrian refugees and the host population in Jordan: a descriptive costing study
Source: Health Policy Plan. 2020 Jul 4;35(8):931–40. doi: 10.1093/heapol/czaa050 (PMC8312704; doi:10.1093/heapol/czaa050)
Supplement: czaa050_Supplementary_Data [file czaa050_supplementary_data.zip › czaa050-Suppl_Data/Supplementary File 4.docx]

Supplementary File 4: Top 20 most costly drug items and proportion of total costs in 2017 (2017 INT$^1^)

| **Item** | **Drug class** | **Consumption^2^** | **Annual cost**  **(2017 INT$)** | **% Total 2017 drug cost** |
| --- | --- | --- | --- | --- |
| 1. INSULIN (MIXTARD) 100 IU, vial | Insulin | 14,399^3^ | 441,640 | 14 |
| 1. STRIP, (glucometer accu check, blood glucose) | Diabetes Equipment | 361,227 | 304,164 | 10 |
| 1. ATORVASTATIN, 20 mg, tab. | Statin cholesterol lowering | 453,786 | 243,896 | 8 |
| 1. SYRINGE, s.u., Luer, insulin, 100 IU/1 ml + fixed needle | Diabetes Equipment | 461,650 | 198,495 | 7 |
| 1. VALSARTAN, 80 mg, tab. | Antihypertensive | 501,630 | 194,115 | 6 |
| 1. ENALAPRIL, 10 mg, tab. | Antihypertensive | 437,117 | 164,452 | 5 |
| 1. SALMETEROL, 50mcg/FLUTICASONE 250mcg, 60 doses, diskus | Asthma/COPD combination inhaler | 1496^4^ | 148,233 | 5 |
| 1. AMLODIPINE, 5 mg, tab. | Antihypertensive | 304,833 | 134,346 | 4 |
| 1. ATORVASTATIN, 80 mg, tab. | Statin cholesterol lowering | 47,993 | 109,484 | 4 |
| 1. ATORVASTATIN, 10 mg, tab. | Statin cholesterol lowering | 315,412 | 108,423 | 4 |
| 1. SALMETEROL, 50mcg/FLUTICASONE 500mcg , 60 doses, diskus | Asthma/COPD combination inhaler | 695^4^ | 93,935 | 3 |
| 1. OMEPRAZOLE, 20 mg, gastro-resistant caps. | Anti-acid/reflux disease | 20,4397 | 83,036 | 3 |
| 1. GLIBENCLAMIDE, 5 mg, breakable tab. | Oral hypoglycaemic | 742,992 | 78,432 | 3 |
| 1. ATENOLOL, 50 mg, tab. | Antihypertensive | 232,239 | 57,581 | 2 |
| 1. METFORMIN hydrochloride, 850 mg, tab. | Oral hypoglycaemic | 639,820 | 54,565 | 2 |
| 1. HYDROCHLOROTHIAZIDE, 25mg, tab. | Antihypertensive | 229,850 | 47,112 | 2 |
| 1. ENALAPRIL maleate, 20 mg, tab. | Antihypertensive | 75,596 | 45,710 | 1 |
| 1. VALSARTAN, 160 mg, tab. | Antihypertensive | 108,345 | 45,034 | 1 |
| 1. ENALAPRIL maleate, 5 mg, tab. | Antihypertensive | 149,569 | 44,212 | 1 |
| 1. RANITIDINE, 75 mg, tab. | Anti-acid/reflux disease | 202,588 | 35,934 | 1 |
| **TOP 20 DRUG ITEMS TOTAL** |  |  | **2,632,798** | **86** |
| **TOTAL 2017 DRUG COSTS** |  |  | **3,049,381** | **100** |

Notes:

^1^Costs are presented in 2017 International Dollars [using Purchasing Power Parity (PPP) to convert JOD and Euro nominal costs into INT$]

^2^Consumption refers to the annual consumption of tablets for each item and reflects the amount of medication dispensed by the pharmacy. It does not necessarily reflect the number of doses prescribed or the number of doses taken by patient, nor does it include wastage.

^3^Insulin consumption refers to the number of vials dispensed per year. Each vial may contain several doses; the total number of doses will depend on the number of units of insulin prescribed to the individual patient.

^4^Combination inhalers for asthma/ COPD contain a month-long course
