# Supplementary material for: Delivering a primary-level non-communicable disease programme for Syrian refugees and the host population in Jordan: a descriptive costing study
Source: Health Policy Plan. 2020 Jul 4;35(8):931–40. doi: 10.1093/heapol/czaa050 (PMC8312704; doi:10.1093/heapol/czaa050)
Supplement: czaa050_Supplementary_Data [file czaa050_supplementary_data.zip › czaa050-Suppl_Data/Supplementary File 5.docx]

*Supplementary file 5. Annual cost per cost level and total financial costs for Irbid NCD Programme for 2015, 2016, 2017, expressed in International dollars.*

| **Year** |  | **2015** | | **2016** | | **2017** | |
| --- | --- | --- | --- | --- | --- | --- | --- |
| **Cost Level** |  | INT $1 | % of total INT $ costs | INT $ | % of total INT$ costs | INT$ | % of total INT$ costs |
| Clinic Level | Capital | 31,223 | 0.7% | 38,491 | 0.6% | 43,199 | 0.6% |
|  | Recurrent (excl HR and drugs) | 777,345 | 18.5% | 647,627 | 10.1% | 626,737 | 9.3% |
|  | HR | 821,830 | 19.5% | 1,234,517 | 19.3% | 1,362,161 | 20.2% |
|  | Drugs | 1,615,967 | 38.4% | 3,008,539 | 47.0% | 3,049,381 | 45.2% |
| **Total Clinic** | | **3,246,365** | **77.2%** | **4,929,175** | **77.0%** | **5,081,478** | **75.4%** |
| Project Level | Capital | 14,512 | 0.3% | 53,849 | 0.8% | 53,374 | 0.8% |
|  | Recurrent (excl HR) | 185,132 | 4.4% | 289,182 | 4.5% | 253,420 | 3.8% |
|  | HR | 424,957 | 10.1% | 769,915 | 12.0% | 805,177 | 11.9% |
| **Total Project** | | **624,600** | **14.8%** | **1,112,946** | **17.4%** | **1,111,971** | **16.5%** |
| Coordination Level | Capital | 2,872 | 0.1% | 8,029 | 0.1% | 10,160 | 0.2% |
|  | Recurrent (excl HR) | 102,815 | 2.4% | 85,514 | 1.3% | 150,485 | 2.2% |
|  | HR | 229,830 | 5.5% | 264,947 | 4.1% | 385,344 | 5.7% |
| **Total Coordination** | | **335,516** | **8.0%** | **358,491** | **5.6%** | **545,989** | **8.1%** |
| **Total Annual Cost** | | **4,206,481** |  | **6,400,611** |  | **6,739,438** |  |

*Notes:*

^1^Costs are presented in 2017 International Dollars [using Purchasing Power Parity (PPP) to convert JOD and Euro nominal costs into INT$]
