# Supplementary material for: Delivering a primary-level non-communicable disease programme for Syrian refugees and the host population in Jordan: a descriptive costing study
Source: Health Policy Plan. 2020 Jul 4;35(8):931–40. doi: 10.1093/heapol/czaa050 (PMC8312704; doi:10.1093/heapol/czaa050)
Supplement: czaa050_Supplementary_Data [file czaa050_supplementary_data.zip › czaa050-Suppl_Data/Supplementary File 6.docx]

Supplementary File 6: Assumptions and cost inputs used for consultation model scenario analyses

| **Data/assumptions** | **Base case** | **Data source/ notes** |
| --- | --- | --- |
| **Potential total cohort** | 4000.0 | NCD Advisor report; interviews with management |
| **Active patients end of 2017** | 3540.0 | Epi results for evaluation |
| **Potential extra capacity in the system at end of 2017** | 460.0 |  |
| **Duration new appt minutes** | 30.0 |  |
| Duration f/u appt minutes | 15.0 |  |
| **Available doctor f/u appointment slots per day (15 min)** | 52.0 | (4 MD x 13 slots @15 min.s);NCD advisor report |
| **Available doctor new appt slots per day (30 min)** | 6.0 | NCD advisor report |
| **Total available doctor 15 minute slots per day** | 64.0 |  |
| **Theoretical number of slots available per month @24 working days/month** | 1536.0 |  |
| Currently |  |  |
| **Actual average follow up consultations per month in 2017** | 2197.375 | MMR 2017 July |
| **Actual avg total nurse f/u per month** | 122.0 | MMR 2017 July |
| **Actual avg total Dr f/u per month** | 1956.0 | MMR 2017 July |
| **Actual Avg Dr f/u per month per doctor** | 489.0 |  |
| **Stable proportion of active cohort** | 0.6 | Based on cohort analysis results |
| **Unstable proportion** | 0.4 |  |
| **Number of stable patients in 2017** | 2124.0 | Epi results for evaluation |
| **Visits / yr unstable pt - in theory** | 12.0 | NCD Guideline |
| **Proposed visits / yr stable pt - in theory** | 4.0 | NCD Guideline |
| **Esti**mates of workload based on task shifting of stable pts to nurses |  |  |
| **Required number of quarterly visits/yr current stable pts** | 8496.0 |  |
| **% DNAs - require rebooking** | 0.05 | NCD advisor report |
| **Required number of 3 monthly visits/month current stable pts** | 743.4 |  |
| **Estimate of proportion stable monthly nurse visits potentially referred to doctor** | 0.1 |  |
| **Unstable patients in 2017** | 1416.0 |  |
| **Number of monthly visits/yr current unstable pts.** | 16992.0 |  |
| **Required number monthly f/u visits/month current unstable pts. incl DNA** | 1486.8 |  |
| **Current rate of new patients per month** | 5.0 |  |
| **Required Dr 15 min appt slots for new pts/ month** | 10.0 |  |
| **Required Dr 15 min appt slots for pt.s referred from nurse** | 74.3 | Based on estimate c26 |
| **Total Dr 15 min slots req'd per month to see unstable + 10% stable + new pts** | 1571.1 |  |
| Work Pattern and Salaries |  |  |
| **Working days per month** | 25.0 |  |
| **Unspecialised Dr annual salary per person** | 17568.0 | JOD; HRCo email |
| **Specialised Dr annual salary per person** | 28212.0 | JOD; HRCo email |
| **Annual insurance per person** | 1036.7 | JOD |
| **Total annual per person unspecialised doctor costs** | 18604.7 |  |
| **Total annual per person specialised doctor costs** | 29248.7 |  |
| **Nurse monthly salary per person** | 9805.609615 | JOD |
| **Nurse annual insurance per person** | 1036.7 | JOD |
| **Total annual per person nurse costs** | 10842.3 |  |
| Per FTE doctor appointment capacity |  |  |
| **Bank holidays per year** | 15.0 | online |
| **Annual leave weeks per year** | 4.0 | assumption |
| **Average sick leave weeks taken per doctor** | 1.0 | assumption based on interviews with management |
| **Annual working weeks if annual leave = 4 weeks/sick leave = 1 week** | 47.0 |  |
| **Working days per week** | 6.0 |  |
| **Total working days per year** | 267.0 |  |
| **Average total working days per month** | 22 | accounting for leave and bank holidays |
| **Working hours per day minus 30 minutes' break time** | 5.5 | based on observation |
| **15m. appt slots per working hour** | 4.0 |  |
| **15m. appt slots per working day per doctor/nurse incl. 30 min break** | 22.0 |  |
| Doctors |  |  |
| **Theoretical 15m. appt slots per month per FTE doctor** | 489.5 |  |
| **Current number of specialist doctors** | 2.0 |  |
| **Ccurrent number of non-specialist doctors** | 2.0 |  |
| **Total monthly doctor 15m. slots available** | 1958.0 |  |
| **Total monthly doctor 15m. slots used currently** | 1966.0 |  |
| **No. FTE Drs needed to manage theoretical total Dr 15 slots required** | 3.2 |  |
| **Round to the nearest 0.5 of a doctor** | 3.5 |  |
| **Minimum of one specialist** | 1 |  |
| **Remaining number of doctors required** | 2.5 |  |
| Nurses |  |  |
| **f/u appt slots per month per FTE nurse** | 489.5 |  |
| **Number of nurses** | 1.0 |  |
| **Ttotal monthly nurse f/u slots** | 489.5 |  |
| **Total monthly nurse 15m. Review slots used currently** | 122.0 |  |
| **No. FTE nurses needed to manage current nurse f/u slots req'd** | 0.2 |  |
| **No. FTE nurses needed to manage theoretical taskshifted nurse f/u slots req'd** | 1.5 |  |
| **Round to nearerst 0.5 of a nurse** | 2 |  |
| Salary costs for providing consultations (JOD) | **Current Model@ current patient load and stability** | |
| **Annual cost for specialist doctor** | 58,497 |  |
| **Annual cost for non specialist doctor** | 37,209 |  |
| **Annual cost for nurses** | 2,702 |  |
| **Total annual cost per consultation model (JOD)** | 98,409 |  |
| **Total annual cost per consultation model (INT$)** | 307,528 |  |

Key: DNA=did not attend; FTE=full time equivalent; f/u=follow up; HRCo=human resources coordinator; INT$=International Dollar; JOD=Jordanian Dinar; MD=medical doctor: MMR=monthly medical report; NCD=non-communicable disease; No.=number
